# Supplementary material for: Projected changes in atmospheric moisture transport contributions associated with climate warming in the North Atlantic
Source: Nat Commun. 2023 Oct 14;14:6476. doi: 10.1038/s41467-023-41915-1 (PMC10576789; doi:10.1038/s41467-023-41915-1)
Supplement: Supplementary file 1 — Supplementary Information [file 41467_2023_41915_MOESM1_ESM.pdf]

Supplementary Information for

Projected changes in atmospheric moisture transport contributions associated with climate warming in the North Atlantic

José C. Fernández-Alvarez[1,2], Albenis Pérez-Alarcón[1,2], Jorge Eiras-Barca[1,3], Stefan Rahimi[4,5], Raquel Nieto[1], Luis Gimeno[1,*]

[1]Centro de Investigación Mariña, Universidade de Vigo, Environmental Physics Laboratory (EPhysLab), Campus As Lagoas s/n, Ourense, 32004, Spain

[2]Departamento de Meteorología, Instituto Superior de Tecnologías y Ciencias Aplicadas, Universidad de La Habana, La Habana, Cuba.

[3]Defense University Center at the Spanish Naval Academy, Group of Applied Mathematics for Defense, Plaza de España s/n 36920 Marín, Spain

[4]Department of Atmospheric Science, University of Wyoming

[5]Center for Climate Science, University of California Los Angeles

* Luis Gimeno

Section 1. Materials and Methodology

Section 1.1 Data description

To obtain the FLEXPART-WRF outputs for our experiments during the historical and future periods (under the SSP5-8.5 scenario), two sets of input data were used:

dynamical downscaled data (using the Weather Research and Forecasting, WRF-ARW, model) from CESM2 outputs and from ERA5 reanalysis (WRF-CESM2 and WRF-ERA5, respectively).

The CESM2 (Community Earth System Model Version 2)[1] data were downloaded from the Earth System Grid Federation (ESGF2) and they were obtained for the native "gn" grid with a resolution of 0.9 x 1.25 (~1°) and presented as an output mesh with 288 × 192 longitude/latitude, 32 vertical levels (top level at 2.25 mb). To force the WRF-ARW model, all CESM2 climatic data were processed to create intermediate files (26 vertical levels) that were used as the initial and boundary conditions. The CESM2 data has been evaluated for representing jet streams and storm tracks, Northern Hemisphere (NH) stationary waves, global divergent circulation, annular modes, the North Atlantic Oscillation and NH winter blocking[2]. CESM2 ranks within the top 10% of CMIP class models with respect to many of these features[2]. CESM2 provides all the necessary variables to force WRF-ARW at a better resolution than other models that which have a resolution of approximately 250 km.

In addition, precipitation in CESM2-based subseasonal forecast systems has been shown to be similar to the one obtained with NOAA CFSv2 model, and slightly lower than the one provided by the ECMWF model[3]. Besides, the North Atlantic Oscilation (NAO) structure in winter and summer is relatively well represented in CESM2 with some minor biases that are quite similar to the rest of the CMIP6 climate models[2]. On the one hand, this implies an adequate representation of the associated precipitation anomalies over the

Mediterranean[4]. According to these authors, many models present inadequacies in the representation, being the amplitude of the precipitation signal too weak, especially in the East. On the other hand, CESM2 has a remarkable representation of the velocity potential of the upper troposphere in both summer and winter. This element is closely related to tropical precipitation and represents a significant forcing of extratropical standing waves[2]. Moreover, it presents improvements in rainfall in regions of great global interest such as the Indian Ocean, East Asia, the tropical Atlantic and the Amazon[2]. Finally, CESM2 has been used to study the sea surface temperature effect increase on future changes in Atmospheric Rivers[5, 6].

The shared socioeconomic pathway (SSP) used in this research was SSP5-8.5[7,8]. SSP5-8.5 is a scenario ("worst-case scenario") that represents emissions high enough to produce a radiative forcing of 8.5 W m^{-2} in 2100 under extreme conditions. This will amplify all signals detected in this analysis.

ERA5[9] is the most recent (5th generation) global atmospheric reanalysis of the European Centre for Medium-Range Weather Forecasts (ECMWF), and it was used to compare fields for the historical period of 1985–2014. WRF-ERA5 outputs were employed to force the FLEXPART-WRF model. These simulations corresponded to the control experiments for evaluating the configuration (see Section 1.4). The advantages of ERA5 are its high resolution (31 km horizontally and 137 vertical levels) and large number of assimilated historical observations. ERA5 significantly improves upon its predecessor, ERA-Interim

reanalysis, particularly with respect to precipitation fields both over extratropical regions and tropical oceanic areas.

Section 1.2 Experimental setup for WRF and FLEXPART-WRF

The parameterisations employed in the WRF-ARW configuration were as follows: the WSM6 microphysics scheme[10], Yonsei University planetary boundary layer (PBL) scheme[11], revised MM5 surface layer scheme[12], United Noah Land Surface Model[13], shortwave and longwave RRTMG schemes[14] and the Kain-Fritsch cumulus scheme[15]. Spectral nudging of waves longer than approximately 1000 km was employed to avoid distortion of the large-scale circulation within the regional model domain due to the interaction between the model solution and lateral boundary conditions[16]. The outputs had 40 vertical layers from the surface to 50 hPa with a horizontal spacing of 20 km and they covered an area of 115.39–42.02°W and 19.41ºS–59.51°N (see Fig. 1). The criteria used to select these parameterisation schemes were that they had been evaluated and employed in several previous investigations involving the WRF-ARW domain[17,18] (see Supplementary Fig. 1). For the WRF simulations, a 1-month spin-up was performed before each year to be simulated, and the restart mode was used when the WRF-ARW was stopped. Finally, the outputs of WRF-ARW had 40 vertical levels in sigma coordinates and 480 × 780 nodes in the output grid (~0.18º). The historical periods, mid-century and end of the century used were 1985-2014, 2036-2065 and 2071-2100, respectively.

For the FLEXPART-WRF[19] configuration, we used Hanna's[20] scheme for turbulence parameterisation with the convection scheme activated. This scheme is based on the boundary layer parameters PBL height, Monin–Obukhov length, convective velocity scale, roughness length and friction velocity[19]. We assumed skewed rather than Gaussian turbulence in the convective PBL. The FLEXPART-WRF has forty levels and 400 × 777 points, where in the output mesh where the particles are released. The outputs had spatial and temporal resolutions of 20 km and 6 h, respectively.

Section 1.3 Moisture sources and sinks used

The North Atlantic Ocean source (NATL; Sup. Fig. 1) is considered to be one of the main global oceanic sources that contributes moisture to continental precipitation[21]. This source contributes to several geographical areas, such as eastern North America, Central America, northern and central South America, Europe and northern Africa. Moreover, the NATL is an important oceanic contributor to the North and South American monsoon systems, as well as to the Atlantic Intertropical Convergence Zone (ITCZ)[22,23]. It shows marked seasonal behaviour; the moisture contribution increases during winter and decreases strongly in summer. Meanwhile, previous studies have not observed changes in its size and position[21].

The Mediterranean Sea (MED; Sup. Fig. 1) plays an important role in its surrounding areas in terms of the transport of atmospheric moisture for precipitation. During the boreal winter, it supplies moisture that generates precipitation in continental

areas located over Europe to the northeast; during the summer, it provides moisture to its surroundings in all directions and extending into northern Europe, northeast Africa and the Middle East[21]. The moisture contribution from the MED is higher in summer and relatively lower in autumn and winter[21]. In particular, the western MED contributes directly to rainfall over the Alpine region and the eastern Iberian Peninsula, and it plays an important role in transporting moisture to northern Africa. Furthermore, the central MED has a substantial impact on rainfall over the Hellenic Peninsula and islands and in the central part of North Africa, and the eastern MED influences the Middle East and Egypt[24].

The Iberian Peninsula (IP; Sup. Fig. 1) is located in southwestern Europe and is surrounded by the Mediterranean Sea to the east and the Atlantic Ocean to the west. It is linked to the European continent in its northeastern corner. The precipitation regime north and west of the IP is strongly affected by the mean annual cycle of the Atlantic storm track and its variability, whereas in the interior and east of the IP, it is strongly affected by large-scale synoptic systems and convective precipitation[25]. The main moisture sources affecting the IP correspond to the tropical-subtropical region of North Atlantic Ocean and the Mediterranean Sea (a more local source).

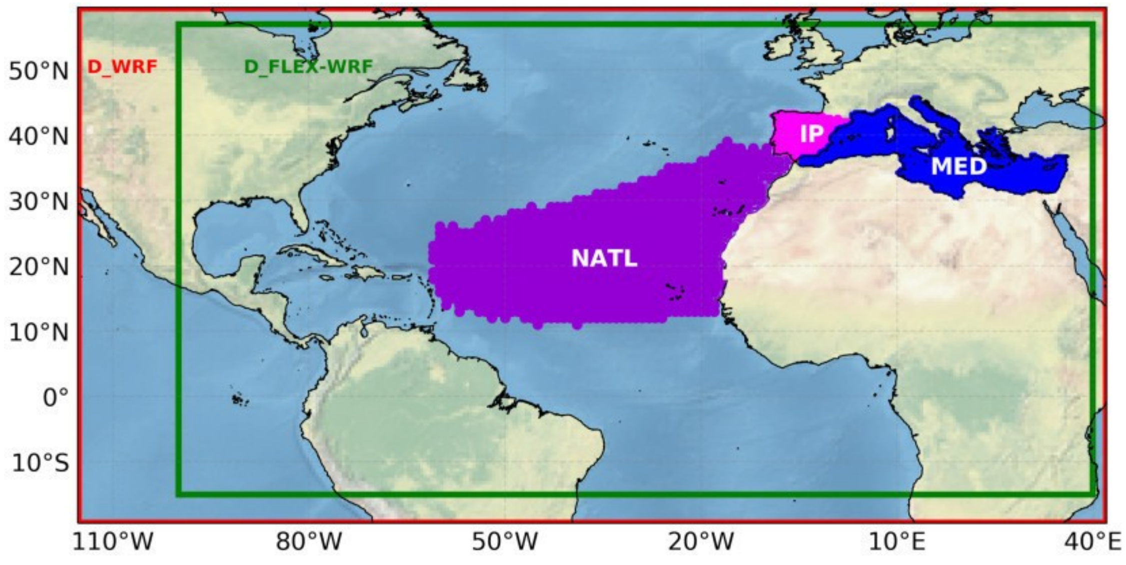

Supplementary Figure 1. Domains and target regions. Domain configuration for WRF-ARW (red) and FLEXPART-WRF (green) simulations. The moisture target regions are shown in blue (MED; Mediterranean Sea), pink (IP; Iberian Peninsula) and dark violet (NATL; North Atlantic moisture source).

Section 1.4 Evaluation of the used configurations

The evaluations were conducted for the reference period (1985–2014) for the boreal seasonal periods from January to March (JFM), April to June (AMJ), July to September (JAS) and October to December (OND), corresponding to winter, spring, summer and autumn, respectively[26], and for the annual scale. The selection of the JFM, AMJ, JAS and OND periods is mainly based on being able to use all the years simulated for the historical, mid- and end-century period. In addition, the consideration of these periods allows us to use the WRF-ARW and FLEXPAT-WRF configuration evaluated with ERA5 for the same study region and the same analyzed periods[27]. Finally, Gimeno et al.[26] used similar periods to study the moisture sources associated with IP, and therefore the ability to make a direct comparison between results is available.

First, the integrated water vapour transport (IVT) with the outputs of WRF-CESM2 and WRF-ERA5 (see Sup. Fig. 10) was evaluated using the following equations,

$$IVT = \sqrt{u_q^2 + v_q^2}, \quad (1)$$

$$u_q = \frac{1}{g} \int_{ps}^{p} uqdp, \quad (2)$$

$$v_q = \frac{1}{g} \int_{ps}^{p} vqdp, \quad (3)$$

where g is gravitational acceleration, q is specific humidity, ps is surface pressure, p is pressure at the top, and u and v are the zonal and meridional winds, respectively[28, 29].

After verifying that the IVT field corresponded with the control experiments, the E-P fields for the moisture sources and sinks were evaluated (see Sup. Fig. 11, 12, 13). The statistics shown in Table 1 were used to evaluate the (E-P) patterns (see Sup. Fig. 14), where x_i and y_i are the simulated (FLEX-WRF) and control (FLEX-ERA5) values, respectively, n is the number of points and $\overline{x_i}$ and $\overline{y_i}$ are the mean values[30].

Supplementary Table 1. Equations for the used statigraphs

**Statigraphs**	**Equation**
Absolute error (MAE)	$MAE = \frac{\sum_{i=1}^{n} \lvert x_i - y_i \rvert}{n}$
Root mean square error (RMSE)	$RMSE = \sqrt{\frac{\sum_{i=1}^{n} (x_i - y_i)^2}{n}}$
Pearson's correlation (R)	$R = \frac{\sum_{i=1}^{n} (x_i - \overline{x_i})(y_i - \overline{y_i})}{\sqrt{\sum_{i=1}^{n} (x_i - \overline{x_i})^2 (y_i - \overline{y_i})^2}}$
Bias (B)	$B = \frac{\sum_{i=1}^{n} (x_i - y_i)}{n}$

Section 2. Supplementary figures

Supplementary figures for: Future projections for precipitation and geopotential height in the Noth Atlantic Ocean from the CESM2 model

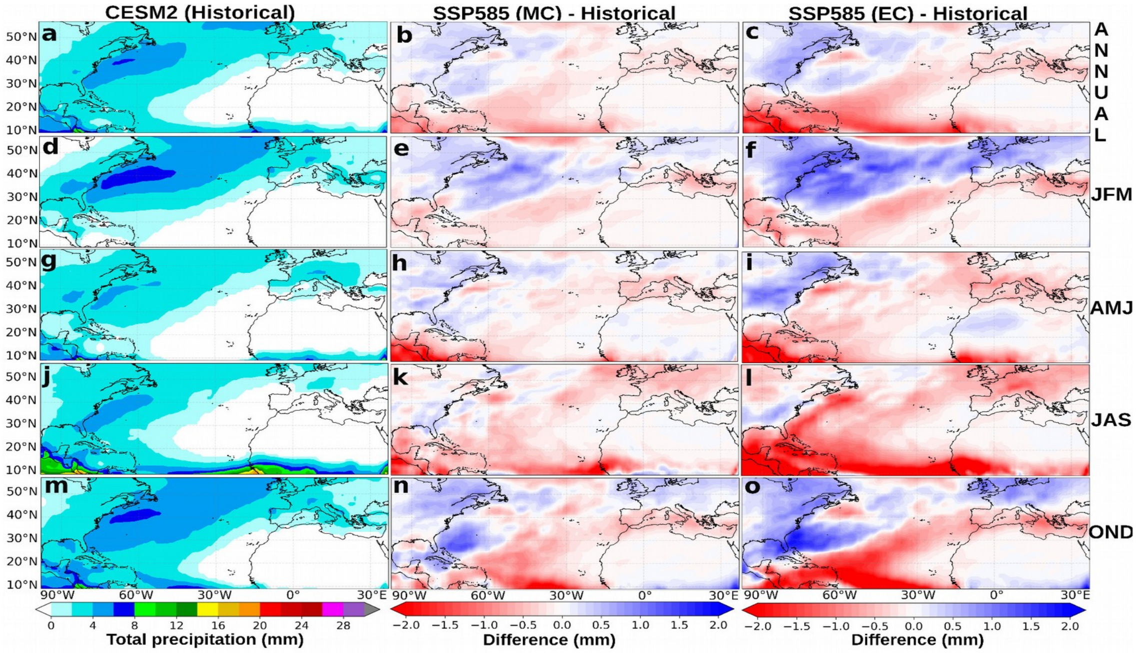

Supplementary Figure 2. Comparison of average seasonal precipitation field between historical period and SSP5-8.5 scenario for CESM2 model. | Precipitation (in mm day^{-1}) for the historical reference period (left column, 1985–2014) and differences with the SSP5-8.5 scenario for the mid- and end 21st century (central, MC, 2036–2065, and right, EC, 2071–2100, respectively).

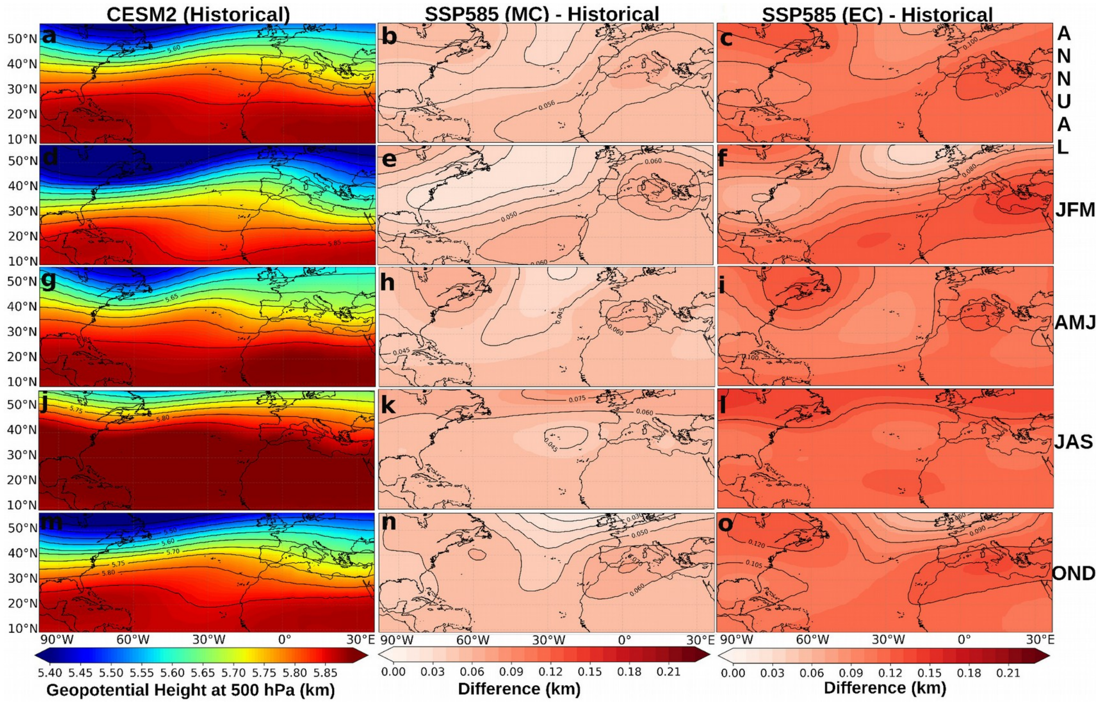

Supplementary Figure 3. Comparison of Geopotential height at 500 hPa field between historical period and SSP5-8.5 scenario for CESM2 model. | Same as Supplementary Figure 2 for geopotential height at 500 hPa (in km).

Supplementary figures and table for: Future projections for integrated water vapour transport (IVT) in the North Atlantic Ocean

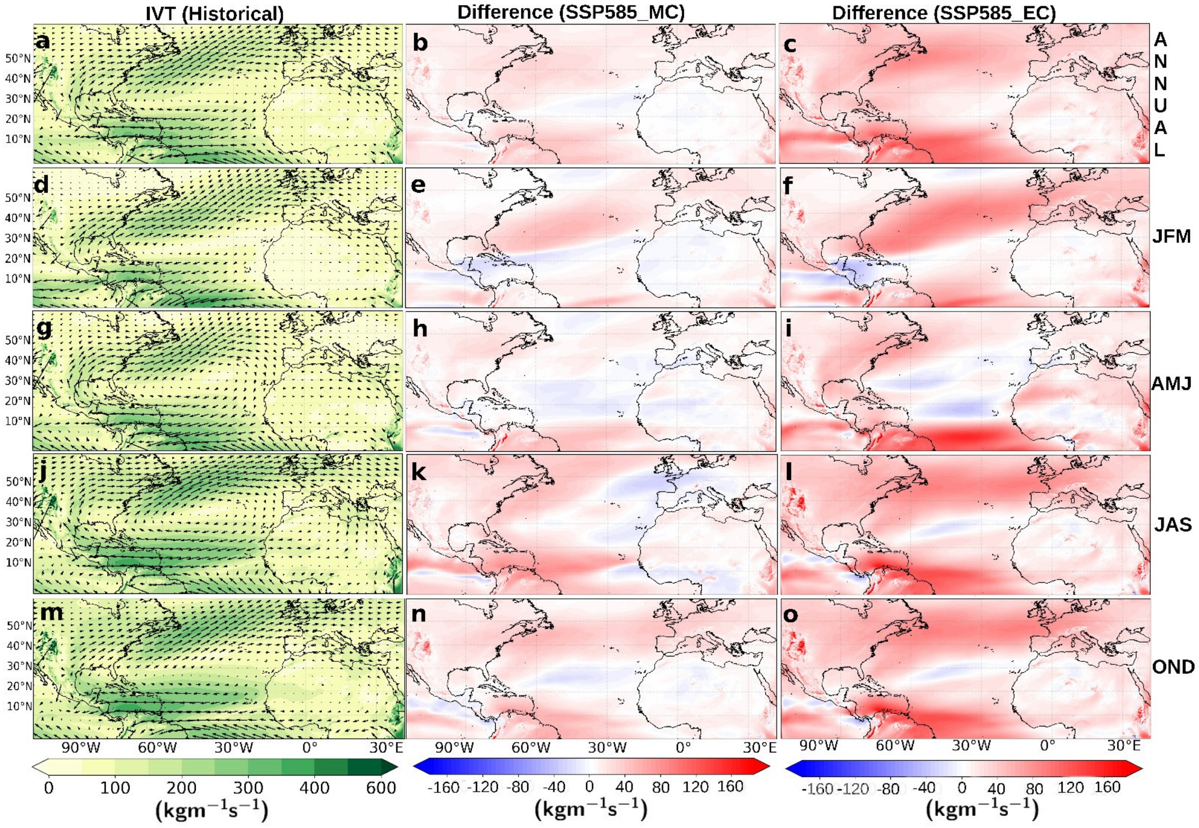

Supplementary Figure 4. Comparison of vertically integrated water vapour transport (IVT) field between historical period and SSP5-8.5 scenario | IVT module (coloured filled, in mm day^{-1}) and direction (arrows, in kg m^{-1} s^{-1}) for the historical reference period (left column, 1985–2014) and differences for the SSP5-8.5 scenario for the mid- and end 21st century (central, MC, 2036–2065, and right, EC, 2071–2100, respectively). The fields displayed from top to bottom correspond to annual, winter, spring, summer and autumn periods (ANNUAL, JFM, AMJ, JAS and OND).

Supplementary Table 2: Mean percentage differences (%) for vertically integrated water vapour transport (IVT) with respect to the historical period considering the SSP5-8.5 scenario | The periods analyzed correspond to the middle (2036–2065, MC) and end of the century (2071–2100, EC).

	Mid-century	End-century
Winter	10.5	24.1
Spring	9.2	21.9
Summer	10.9	27.6
Autumn	14.1	30.1
Annual	11.2	25.9

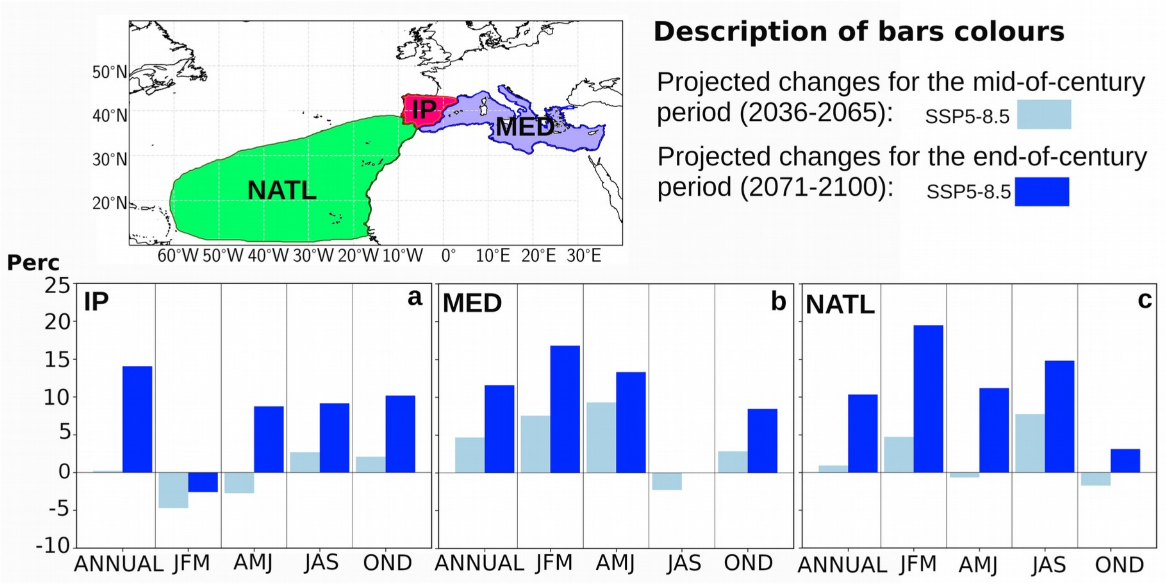

Supplementary Figure 5. Future changes in the yearly and seasonal variability for the relative contribution (in %) of the moisture sources to the Iberian Peninsula | Relative changes in the standard deviation of the time series are shown for Mediterranean Sea (MED), North Atlantic Ocean (NATL) and the precipitation recycling processes (PRPs) over the four seasons as well as in annual terms both for middle (2036–2065, MC) and end of the century (2071–2100, EC).

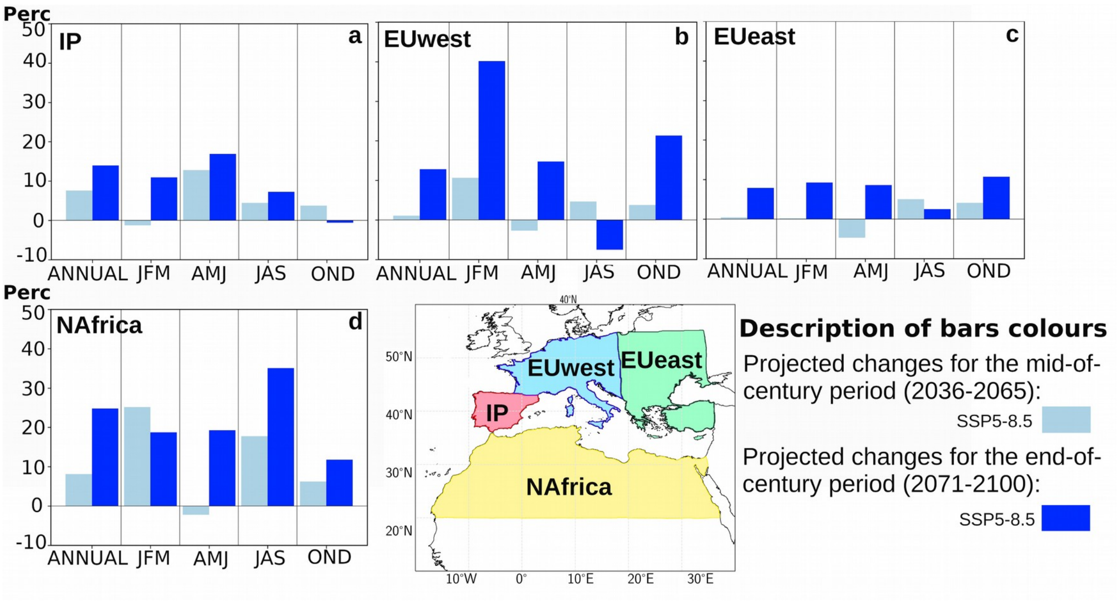

Supplementary Figure 6. Future changes in the yearly and seasonal variability for the relative contribution of precipitation (in %) to moisture sinks from the Mediterranean source | Relative changes in the standard deviation of the time series are shown for Western Europe (EUwest), Eastern Europe (EUeast) and North Africa (Nafrica) and Iberian Peninsula (IP) associated with the Mediterranean sea (MED) source.

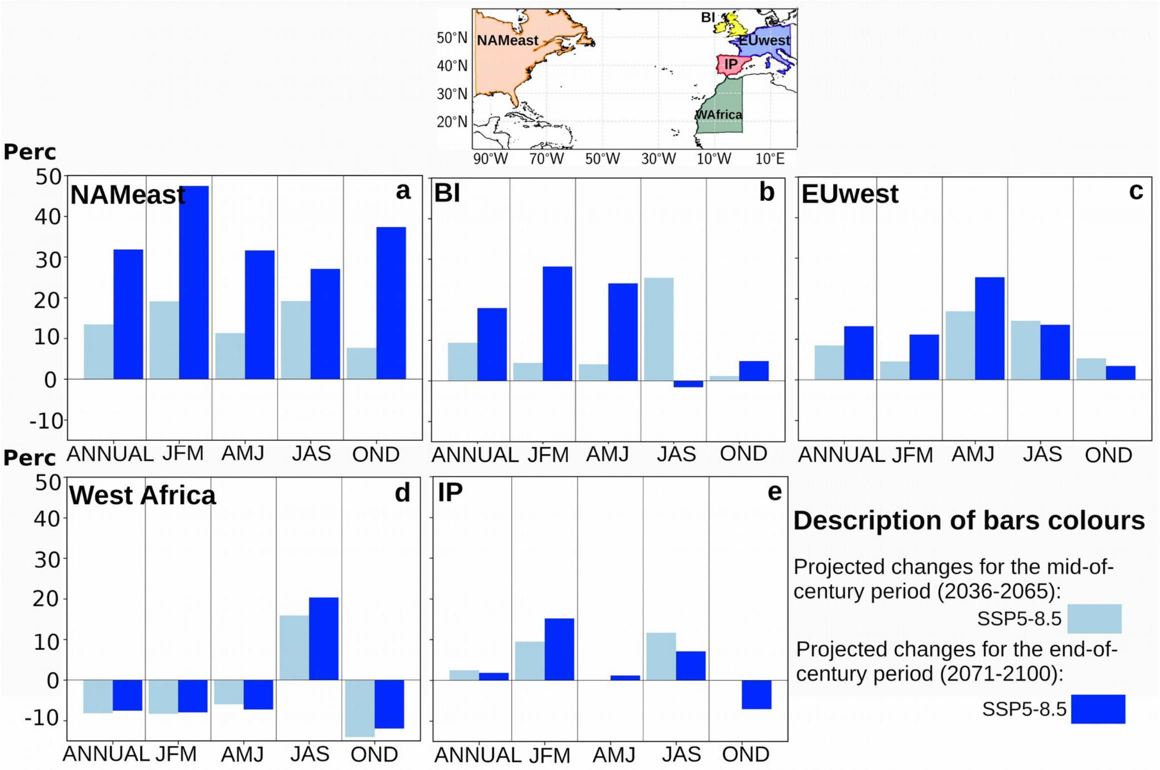

Supplementary Figure 7. Future changes in the yearly and seasonal variability for the the relative contribution of precipitation (in %) to moisture sinks from the North Atlantic source | Relative changes in the standard deviation of the time series are shown for British Isles (BI), European West Coast (EUwest), Iberian Peninsula (IP), West Africa (WAfrica) and the North American East Coast (NAMeast) associated with the North Atlantic Ocean (NATL) source.

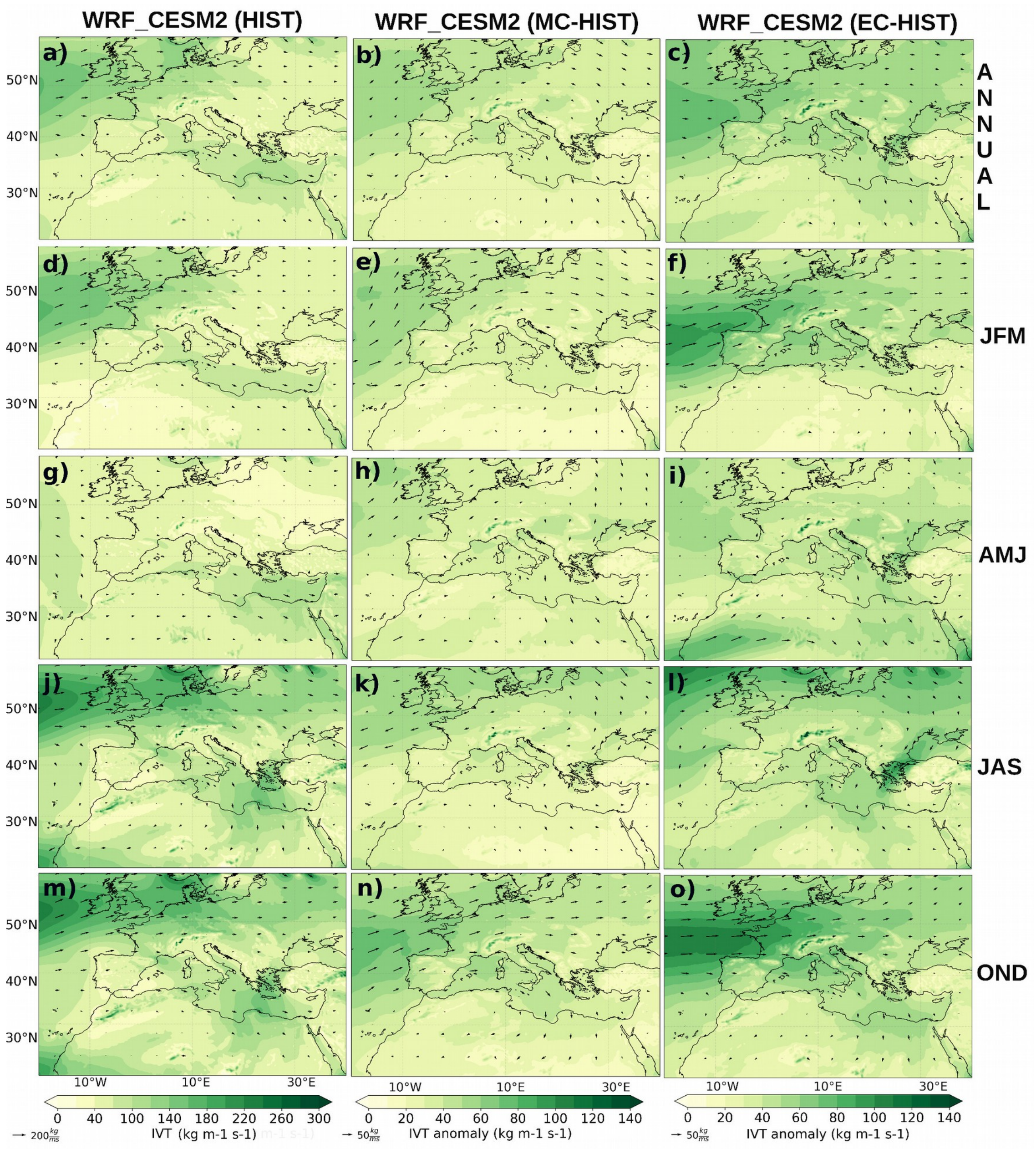

Supplementary Figure 8. Comparison of vertically integrated water vapour transport (IVT) field between historical period and its anomalies with scenario SSP5-8.5 for MC and EC focused on the Mediterranean sea (MED) | IVT module (coloured filled, in mm day^{-1}) and direction (arrows, in kg m^{-1} s^{-1}) for the historical reference period (left column, 1985–2014) and anomalies for the SSP5-8.5 scenario for the mid- and end 21st century (central, MC, 2036–2065, and right, EC, 2071–2100, respectively). The fields displayed from top to bottom correspond to annual, winter, spring, summer and autumn periods (ANNUAL, JFM, AMJ, JAS and OND).

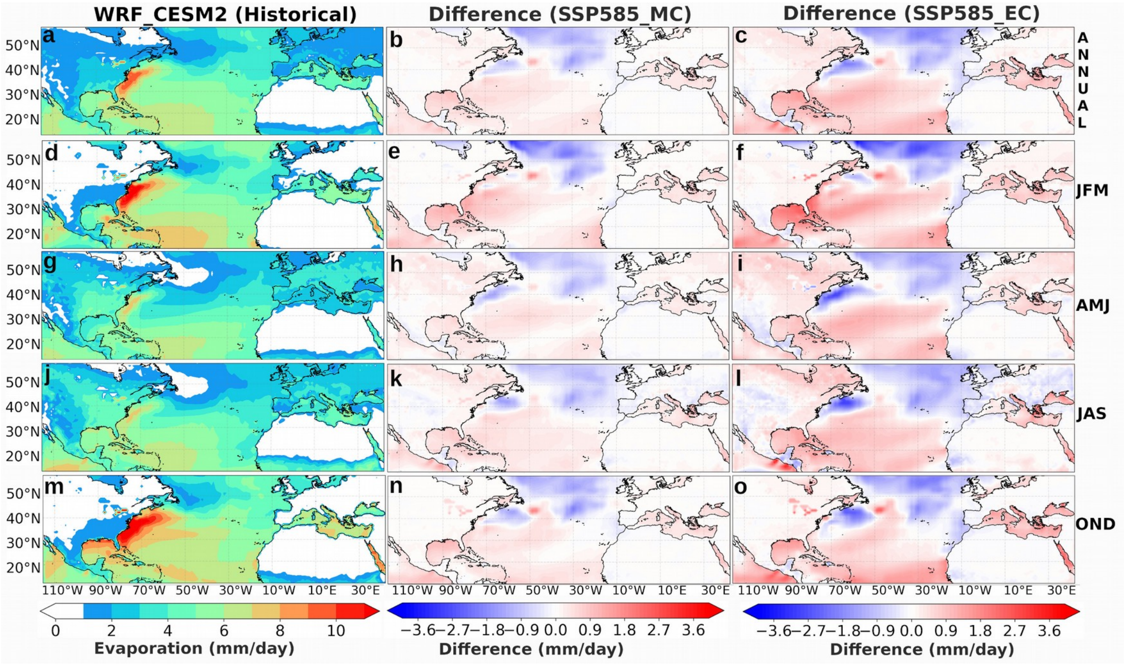

Supplementary Figure 9. Comparison of evaporation (E) field between historical period and SSP5-8.5 scenario focused on the North Atlantic and Mediterranean Sea | E field (coloured filled, in mm day^{-1}) for the historical reference period (left column, 1985–2014) and differences for the SSP5-8.5 scenario for the mid- and end 21st century (central, MC, 2036–2065, and right, EC, 2071–2100, respectively). The fields displayed from top to bottom correspond to annual, winter, spring, summer and autumn periods (ANNUAL, JFM, AMJ, JAS and OND).

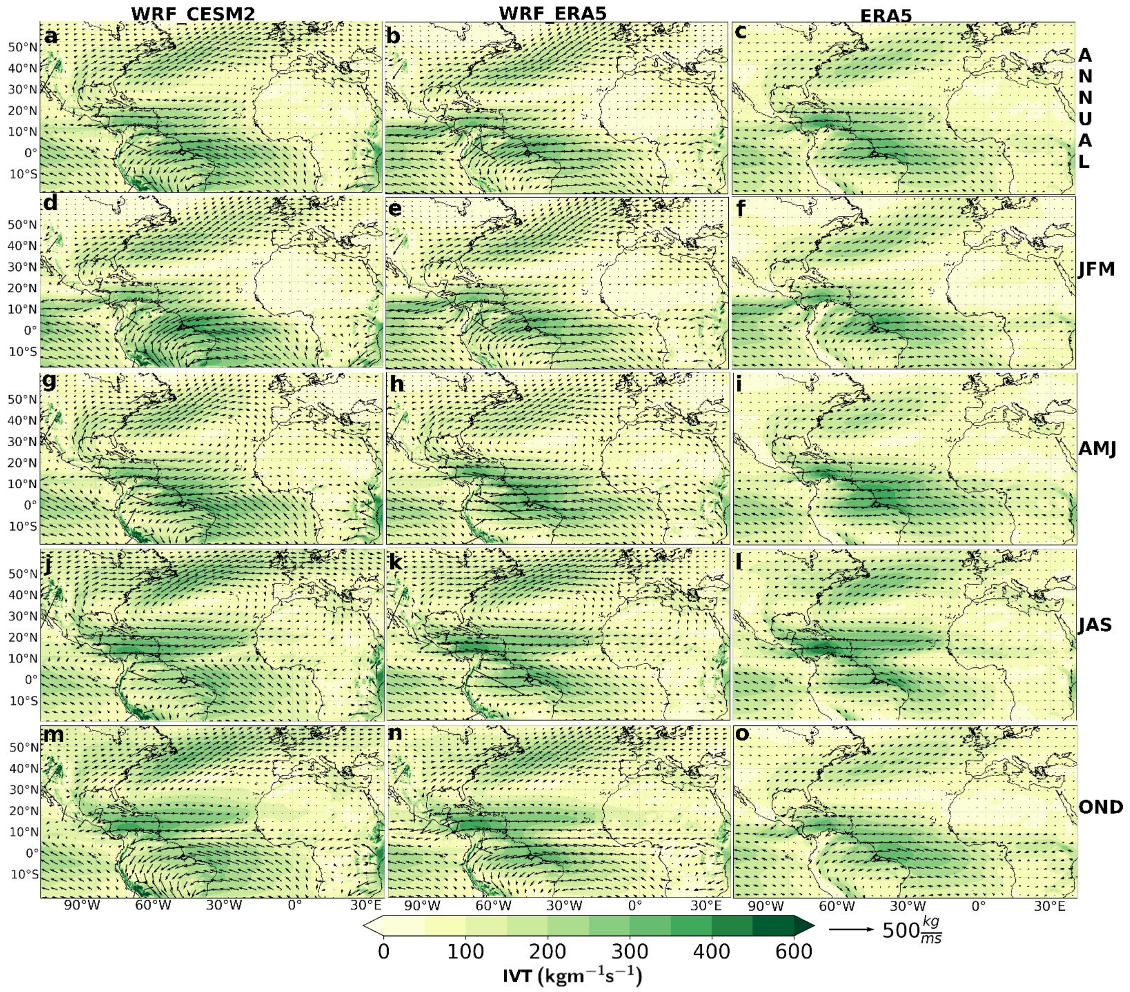

Supplementary Figure 10. Comparison of vertically integrated water vapour transport (IVT) field between WRF-CESM2 and WRF-ERA5 | IVT module (contours) and direction (arrows) for WRF-CESM2 (left column), WRF-ERA5 (central) and ERA5 (right) in the period 1985–2014 (kg m^{-1} s^{-1}). The fields displayed from top to bottom correspond to annual, winter, spring, summer and autumn periods (ANNUAL, JFM, AMJ, JAS and OND).

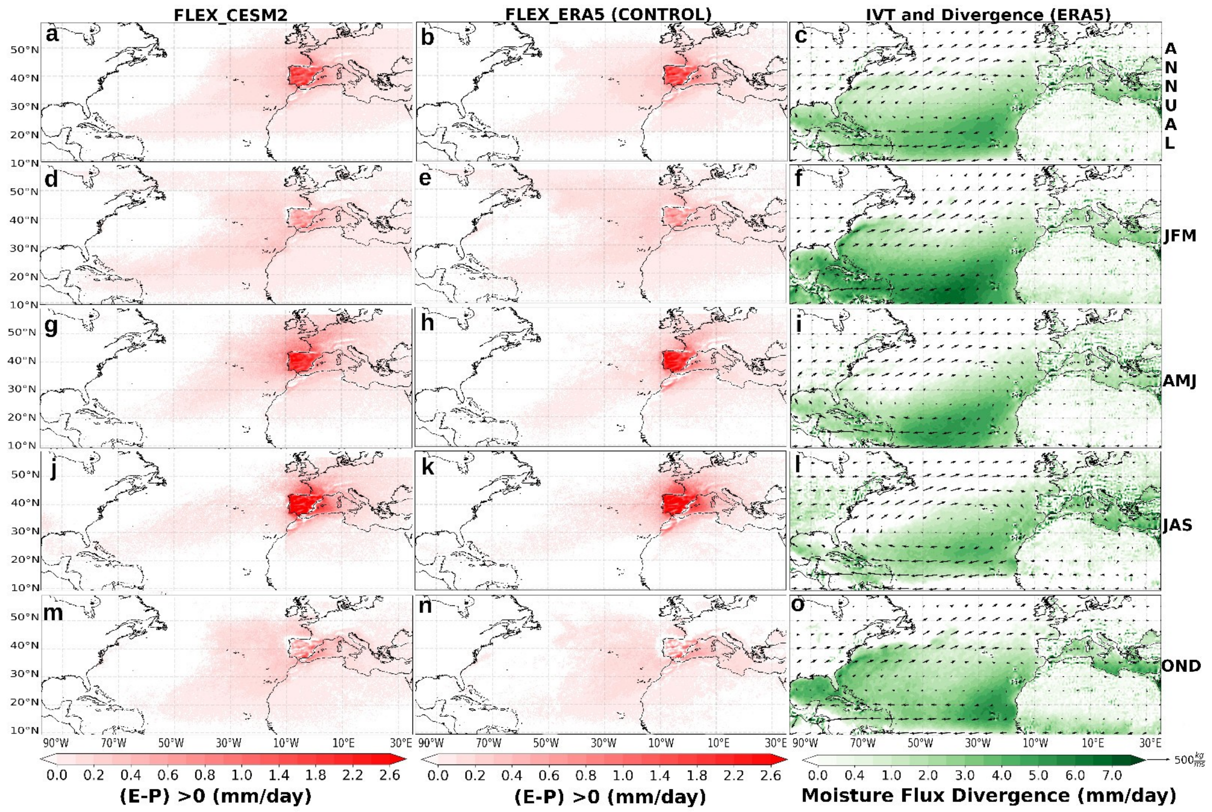

Supplementary Figure 11. Moisture source fields for the Iberian Peninsula in the historical period | Moisture sources fields for the Iberian Peninsula (in mm day^{-1}) for FLEX-CESM2 (left) and FLEX-ERA5 (right), and the vertically integrated water vapour transport (IVT) field and its divergence from ERA5 (right, vectors in vectors in kg m^{-1} s^{-1} and coloured field in mm day^{-1}, respectively) during the historical period (1985-2014). The fields displayed from top to bottom correspond to annual, winter, spring, summer and autumn periods (ANNUAL, JFM, AMJ, JAS and OND).

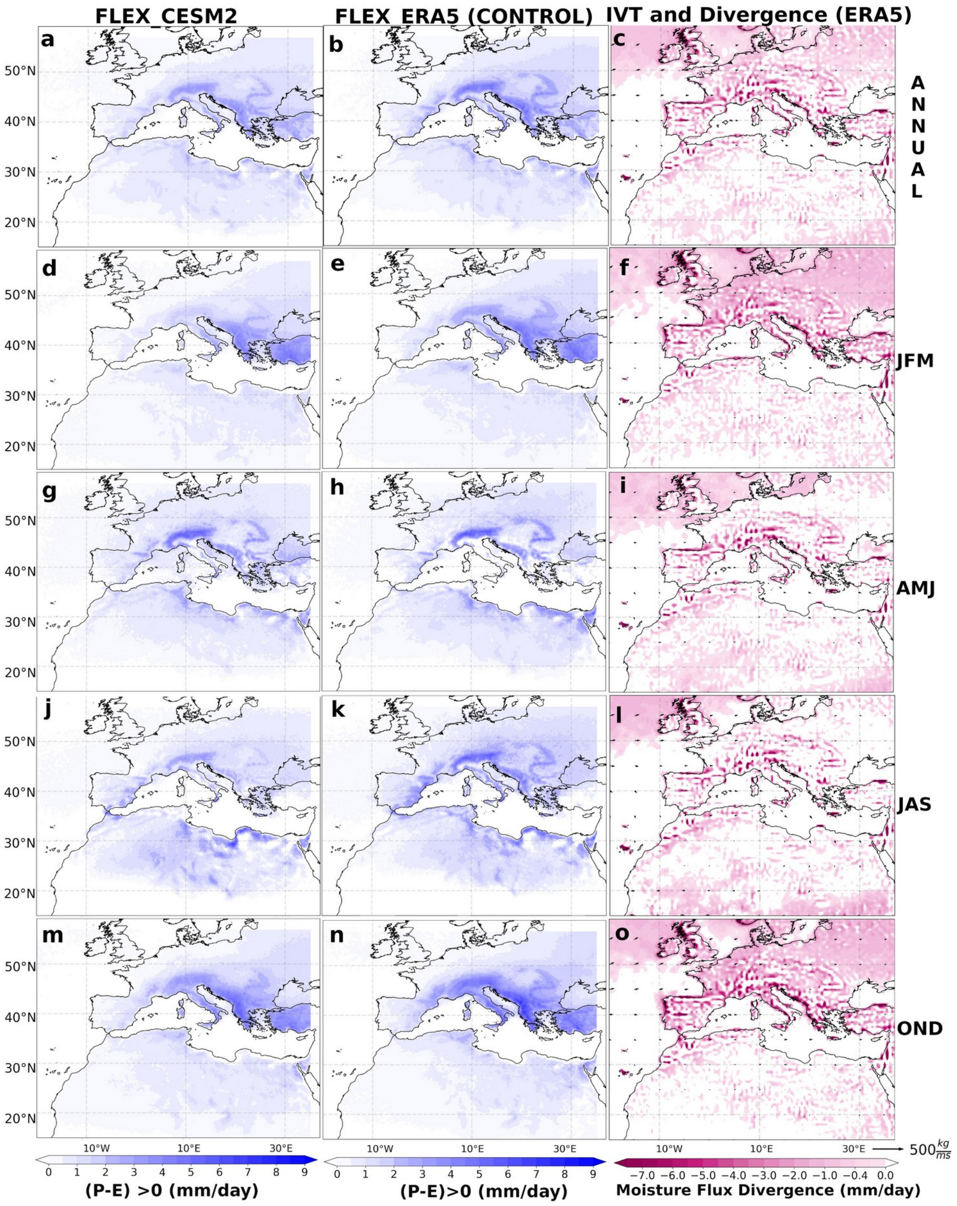

Supplementary Figure 12. Moisture sink fields for the Mediterranean source in the historical period | Moisture sink fields for the Mediterranean Sea (in mm day^{-1}) for FLEX-CESM2 (left) and FLEX-ERA5 (right), and the vertically integrated water vapour transport (IVT) field and its divergence from ERA5 (right, vectors in vectors in kg m^{-1} s^{-1} and coloured field in mm day^{-1}, respectively) during the historical period (1985-2014). The fields displayed from top to bottom correspond to annual, winter, spring, summer and autumn periods (ANNUAL, JFM, AMJ, JAS and OND).

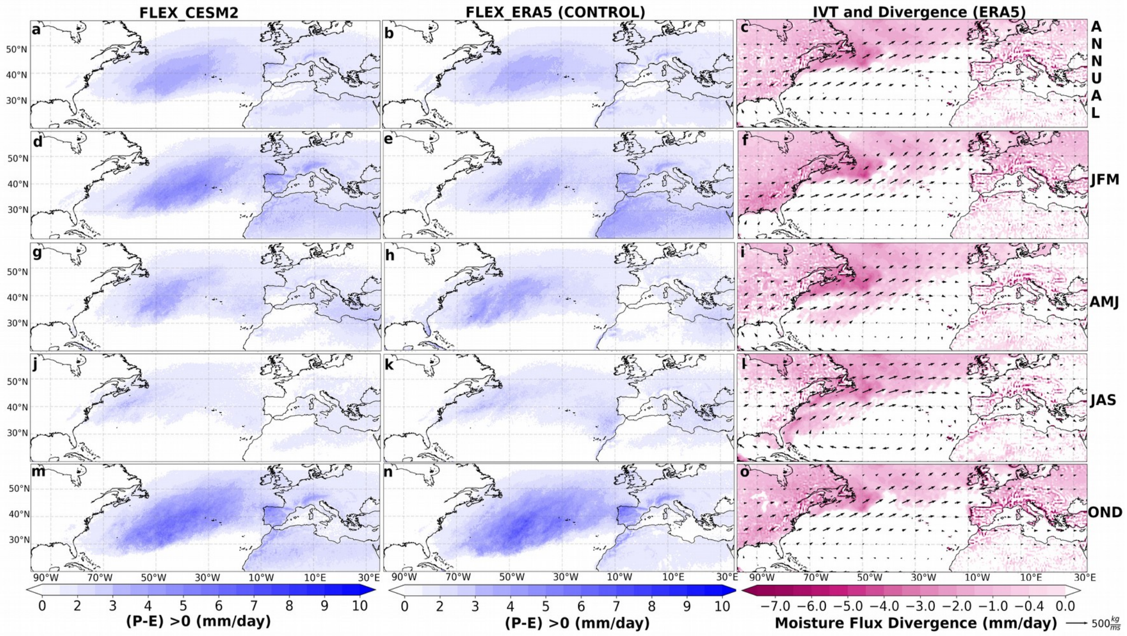

Supplementary Figure 13. Moisture sink fields for the North Atlantic source in the historical period. | Moisture sink fields for the North Atlantic (in mm day^{-1}) for FLEX-CESM2 (left) and FLEX-ERA5 (right), and the vertically integrated water vapour transport (IVT) field and its divergence from ERA5 (right, vectors in vectors in kg m^{-1} s^{-1} and coloured field in mm day^{-1}, respectively) during the historical period (1985-2014). The fields displayed from top to bottom correspond to annual, winter, spring, summer and autumn periods (ANNUAL, JFM, AMJ, JAS and OND).

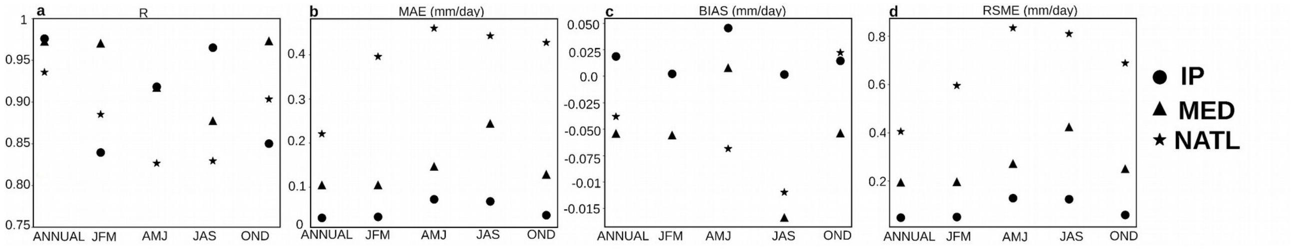

Supplementary Figure 14. Evaluation of FLEX-CESM2 configuration in the historical period. | Statigraphs used for evaluating moisture sources and sinks for target regions: Iberian Peninsula (IP), Mediterranean Sea (MED) and North Atlantic Ocean (NATL). The graphs shown correspond to: Pearson's correlation (R) (a), Absolute error (MAE) (b), Bias (BIAS) (c), and Root mean square error (RSME) (d). Period: 1985–2014.

Section 3. Comparison of results for WRF-CESM2 model vs Ensemble models database

The CESM2 model is considered warm since it reaches ~4ºC in the period 2060-2079 under the SSP5-8.5 scenario[31]. In order to identify if this behavior can influence the results, the main simulations in this manuscript are compared with those obtained using the outputs from the Bias-corrected CMIP6 global database[32] (WRF-ENS) as forcing data. For this comparison, we used a 5-year period (Historical: 2010-2014, MC: 2049-2053, EC: 2096-2100). These data are a bias-corrected global database based on 18 models from the CMIP6 and the European Center for Medium-Range Weather Forecasts Reanalysis 5 (ERA5) database. The bias-correction of these outputs is based on ERA5 mean climate and interannual variance, with a non-linear trend from the ensemble mean of the 18 CMIP6 models. The dataset spans the historical time period 1979–2014 and future scenarios for 2015–2100 with a horizontal grid spacing of (1.25° × 1.25°) at six-hourly intervals.

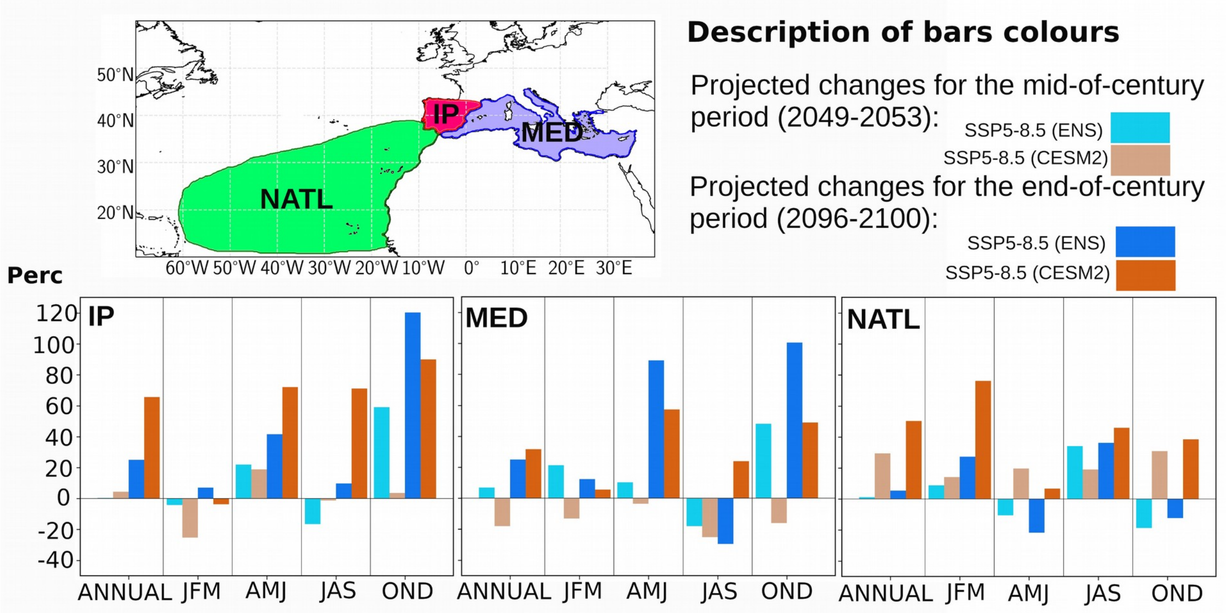

Supplementary Figure 15. Comparison of the future moisture contribution changes for the Iberian Peninsula for WRF-CESM2 vs WRF-ENS. Percentage values of future moisture contribution changes corresponding to the: berian Peninsula (IP), Mediterranean Sea (MED) and North Atlantic Ocean (NATL). The periods are annual, winter, spring, summer and autumn periods (ANNUAL, JFM, AMJ, JAS and OND) and the SSP5-8.5 scenario. Blue and red colours correspond to the WRF-ENS and WRF-CESM2, respectively.

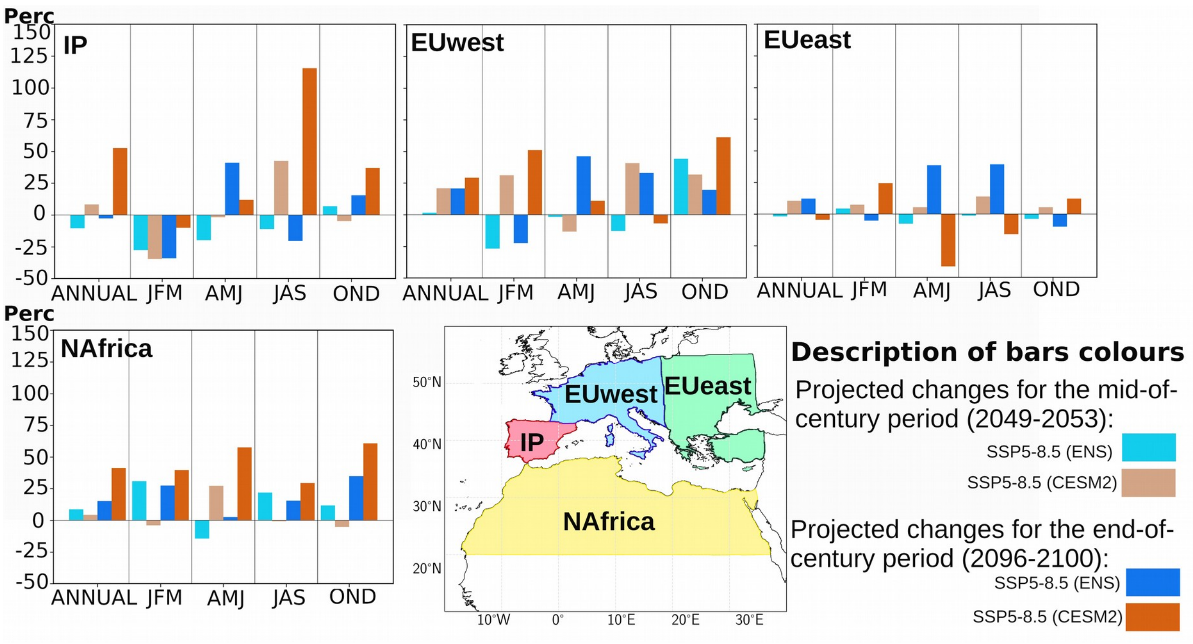

Supplementary Figure 16. Future changes in precipitation contribution associated with the Mediterranean source for WRF-CESM2 vs WRF-ENS. Percentage projected future changes in precipitation contribution over: Western Europe (EUwest), Eastern Europe (EUeast) and North Africa (Nafrica) and Iberian Peninsula (IP) associated with the Mediterranean sea (MED) source**.** The periods are annual, winter, spring, summer and autumn periods (ANNUAL, JFM, AMJ, JAS and OND) and the SSP5-8.5 scenario. Blue and red colours correspond to the WRF-ENS and WRF-CESM2, respectively.

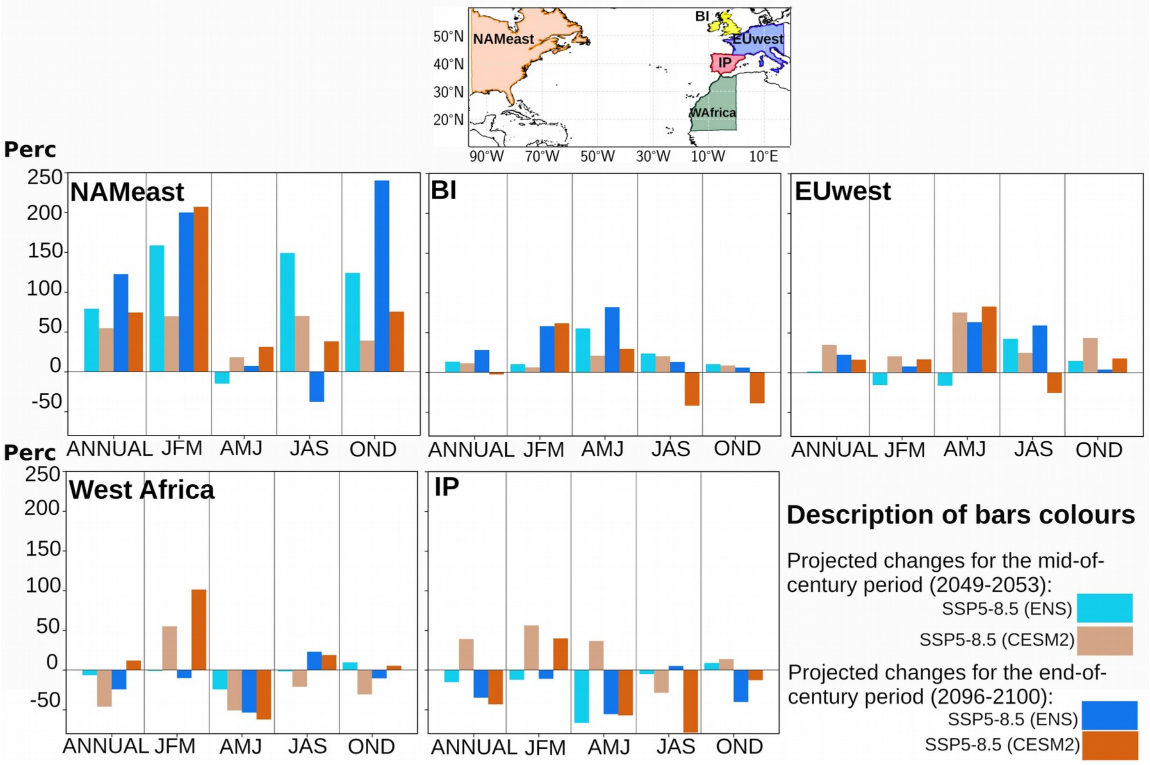

Supplementary Figure 17. Future changes in precipitation contributions from the North Atlantic source for WRF-CESM2 vs WRF-ENS. Percentage future changes in the precipitation contribution over: British Isles (BI), European West Coast (EUwest), Iberian Peninsula (IP), West Africa (WAfrica) and the North American East Coast (NAMeast) associated with the North Atlantic Ocean (NATL) source. The periods are annual, winter, spring, summer and autumn periods (ANNUAL, JFM, AMJ, JAS and OND) and the SSP5-8.5 scenario. Blue and red colours correspond to the WRF-ENS and WRF-CESM2, respectively.

Supplementary References

1- Danabasoglu, G., Lamarque, J.-F., Bacmeister, J., Bailey, D. A., DuVivier, A. K., Edwards, J., et al. The Community Earth System Model Version 2 (CESM2). *J. Adv. Model. Earth Syst.* **12,** e2019MS001916 (2020).

2- Simpson, I. R., Bacmeister, J., Neale, R. B., Hannay, C., Gettelman, A., Garcia, R. R., et al. An evaluation of the large-scale atmospheric circulation and its variability in CESM2 and other CMIP models. *J. Geophys. Res.* **125,** e2020JD032835 (2020).

3- Richter, J. H. et al. Subseasonal Earth system prediction with CESM2. *Weather Forecast.* **37**, 797-815 (2022).

4- Bladè, I., Fortuny, D., van Oldernborgh, G. J., Liebmann, B. The summer North Atlantic Oscillation in CMIP3 models and related uncertainties in projected summer drying in Europe. *J. Geophys. Res.* **117**, D166104 (2012).

5- McClenny, E. E., Ullrich, P. A., Grotjahn, R. Sensitivity of atmospheric river vapor transport and precipitation to uniform sea surface temperature increases. *J. Geophys. Res. Atmos.* **125**, e2020JD033421 (2020).

6- McClenny, E. E. Using Simpler Models to Understand Atmospheric River Responses to Sea-Surface Temperature Increases (Doctoral dissertation, University of California, Davis) (2021).

7- O'Neill B. C. et al. The scenario model intercomparison project (ScenarioMIP) for CMIP6. *Geosci. Model Dev.* **9**, 3461–3482 (2016)

8- Riahi K. et al. The shared socioeconomic pathways and their energy, land use, and greenhouse gas emissions implications: an overview. *Glob. Environ. Change* **42**, 153-168 (2017).

9- Hersbach, H., Bell, B., Berrisford, P., Hirahara, S., Horányi, A., Muñoz-Sabater, J., et al. The ERA5 global reanalysis. *Q. J. R. Meteorol. Soc.* **146,** 1999–2049 (2020).

10- Hong, S. Y., & Lim, J. O. J. The WRF single-moment 6-class microphysics scheme (WSM6). *Journal of the Korean Meteorological Society* **42,** 129–151 (2006).

11- Hong, S. Y., Noh, Y., & Dudhia, J. A new vertical diffusion package with an explicit treatment of entrainment processes. *Mon. Weather Rev.* **134,** 2318–2341 (2006).

12- Jimenez, P. A., Dudhia, J., Gonzalez-Rouco, J. F., Navarro, J., Montavez, J. P., & Garcia–Bustamante, E. A revised scheme for the WRF surface layer formulation. *Mon. Weather Rev.* **140,** 898–918 (2012).

13- Tewari, M., Chen, F., Wang, W., Dudhia, J., LeMone, M., Mitchell, K., et al. Implementation and verification of the unified Noah land surface model in the WRF model, 20th Conference on Weather Analysis and Forecasting/16th Conference on Numerical Weather Prediction, Seattle, WA (2004).

14- Iacono, M. J., Delamere, J. S., Mlawer, E. J., Shephard, M. W., Clough, S. A., & Collins, W. D. Radiative forcing by long–lived greenhouse gases: Calculations with the AER radiative transfer models. *J. Geophys. Res.* **113**, D13103 (2008).

15- Kain, J. S. The Kain–Fritsch Convective Parameterization: An Update. *J. Appl. Meteorol.* **43,** 170-181 (2004).

16- Miguez-Macho, G., Stenchikov, G. L., & Robock, A. Spectral nudging to eliminate the effects of domain position and geometry in regional climate model simulations. *J. Geophys. Res. Atmos.* **109,** 1–15 (2004).

17- Insua-Castro, D. & Miguez-Macho, G. A new moisture tagging capability in the Weather Research and Forecasting model: formulation, validation and application to the 2014 Great Lake-effect snowstorm. *Earth. Syst. Dyn.* **9,** 167–185 (2018).

18- Insua-Castro, D., Miguez-Macho, G. & Llasat, M. C. Local and remote moisture sources for extreme precipitation: a study of the two catastrophic 1982 western Mediterranean episodes. *Hydrol. Earth Syst. Sci.* **23**, 3885–3900 (2019).

19- Brioude, J., et al. The Lagrangian particle dispersion model FLEXPART-WRF version 3.1. *Geosci. Model Dev.* **6,** 1889–1904 (2013).

20- Hanna, S. R. Applications in air pollution modeling, in: Atmospheric Turbulence and Air Pollution Modelling. Reidel Publishing Company, Dordrecht, Holland, 275–310 (1982).

21- Gimeno, L., Drumond, A., Nieto, R., Trigo, R. M. & Stohl, A. On the origin of continental precipitation. *Geophys. Res. Lett.* **37,** L13804 (2010).

22- Castillo, R., Nieto, R., Drumond, A. & Gimeno, L. Estimating the Temporal Domain when the Discount of the Net Evaporation Term Affects the Resulting Net Precipitation Pattern in the Moisture Budget Using a 3-D Lagrangian Approach. *PLoS ONE* **9,** e99046 (2014).

23- Drumond, A., Nieto, R., & Gimeno, L. On the contribution of the Tropical Western Hemisphere Warm Pool source of moisture to the Northern Hemisphere precipitation through a Lagrangian approach, *J. Geophys.* **116,** D00Q04 (2011).

24- Nieto, R., Gimeno, L., Drumond, A. & Hernandez, E. A Lagrangian identification of the main moisture sources and sinks affecting the Mediterranean area. *WSEAS Trans. Environ. Dev.* **6,** 365-374 (2010).

25- Trigo, I. F., Davies, T. D. & Bigg, G. R. Objective climatology of cyclones in the Mediterranean region. *J. Climate* **12,** 1685–1696 (1999).

26- Gimeno, L., Nieto, R., Trigo, R., Vicente-Serrano, S.M. & López-Moreno, J. I. Where does the Iberian Peninsula moisture comme from? An answer based on a Lagrangian approach. *J. Hydrometeorol.* **11,** 421-336 (2010).

27- Fernández-Alvarez, J. C., Vázquez M., Pérez-Alarcón A., Nieto R., Gimeno L. Comparison of moisture sources and sinks estimated with different versions of FLEXPART and FLEXPART-WRF models forced with ECMWF reanalysis data. (2022)

28- Lavers, D. A., Villarini, G., Allan, R. P., Wood, E. F., & Wade, A. J. The detection of atmospheric rivers in atmospheric reanalyses and their links to British winter floods and the large-scale climatic circulation. *J. Geophys. Res.* **117,** D20106 (2012).

29- Lavers, D. A., Villarini, G., Allan, R. P., Wood, E. F., & Wade, A. J. The detection of atmospheric rivers in atmospheric reanalyses and their links to British winter floods and the large-scale climatic circulation. *J. Geophys. Res.* **117**, D20106 (2012).

30- Brown B., Ebert E., Fowler T., Gilleland E., Kucera P., Wilson L. Verification methods for tropical cyclone forecasts. WMO Technical Document WWRP 2013-7, 7(November) (2013).

31- Seneviratne, S. I., Hauser, M. Regional Climate Sensitivity of Climate Extremes in CMIP6 Versus CMIP5 Multimodel Ensembles. *Earths Future* **8**, e2019EF001474 (2020).

32- Xu, Z., Han, Y., Tam, C. Y., Yang, Z. L., Fu, C. Bias-corrected CMIP6 global dataset for dynamical downscaling of the historical and future climate (1979–2100). *Scientific Data* **8**, 1-11 (2021).
